# 

**Published:** 1895-12

**Authors:** 


### CONTENTS FOR DECEMBER, 1895.

| ORIGINAL COMMUNICATIONS. | PAGE. |
|---|---|
| Treatment of Headache. By FLOYD S. CREGO, M. D. | 369 |
| Fracture of the Greater Tuberosity of the Humerus, with the Report of a Case. By A. L. HALL, M. D. | 379 |
| Aural, Nasal and Laryngeal Tuberculosis—with Special Reference to the Adirondacks as a Winter Health Resort. By SARGENT F. SNOW, M. D. | 385 |
| The Decadence of Opium Addiction. By WENDELL REBER, M. D. | 392 |
| Mydriatics in Refraction. By RICHARD H. SATTERLEE, M. D. | 394 |
| Gastrostomy in the Seventeenth Century. By F. G. MOEHLAU, M. D. | 395 |
| Note on Posterior Nasal Tampon. By JOHN T. PITKIN, M. D. | 397 |
| **SOCIETY PROCEEDINGS.** | |
| Medical Association of Central New York | 398 |
| **PROGRESS IN MEDICAL SCIENCE.** | |
| Laryngology | 401 |
| State Medical Examinations | 407 |
| Public Health, Hygiene and Bacteriology | 415 |
| **CORRESPONDENCE.** | |
| Criminal Responsibility | 416 |
| Three Children at One Birth | 417 |
| **EDITORIAL.** | |
| The Economics of Municipal Charity | 419 |
| Topics of the Month | 421 |
| Obituary | 423 |
| Personal | 427 |
| Society Meetings | 428 |
| What Our Contemporaries Think of Us | 430 |
| Reviews | 434 |
| Literary Notes | 446 |

